# High risk for human exposure to Rift Valley fever virus in communities living along livestock movement routes: A cross-sectional survey in Kenya

**DOI:** 10.1371/journal.pntd.0007979

**Published:** 2020-02-21

**Authors:** Caroline Tigoi, Rosemary Sang, Edith Chepkorir, Benedict Orindi, Samuel Okello Arum, Francis Mulwa, Gladys Mosomtai, Samson Limbaso, Osama A. Hassan, Zephania Irura, Clas Ahlm, Magnus Evander

**Affiliations:** 1 International Centre of Insect Physiology and Ecology, Nairobi, Kenya; 2 Center for Virus Research, Kenya Medical Research Institute, Nairobi, Kenya; 3 Department of Clinical Microbiology, Virology, Umeå University, Umeå, Sweden; 4 Ministry of Public Health and Sanitation, Nairobi, Kenya; 5 Department of Clinical Microbiology, Infection and Immunology, Umeå University, Umeå, Sweden; University of California, Davis, UNITED STATES

## Abstract

**Introduction:**

Multiple outbreaks of Rift Valley Fever (RVF) with devastating effects have occurred in East Africa. These outbreaks cause disease in both livestock and humans and affect poor households most severely. Communities living in areas practicing nomadic livestock movement may be at higher risk of infection. This study sought to i) determine the human exposure to Rift Valley fever virus (RVFV) in populations living within nomadic animal movement routes in Kenya; and ii) identify risk factors for RVFV infection in these communities.

**Methods:**

A cross-sectional descriptive study design was used. Samples were collected from the year 2014 to 2015 in a community-based sampling exercise involving healthy individuals aged ≥18 years from Isiolo, Tana River, and Garissa counties. In total, 1210 samples were screened by ELISA for the presence of immunoglobulin IgM and IgG antibodies against RVFV. Positive results were confirmed by plaque reduction neutralization test.

**Results:**

Overall, IgM and IgG prevalence for all sites combined was 1.4% (95% CI 0.8–2.3%) and 36.4% (95% CI 33.8–39.2%), respectively. Isiolo County recorded a non-significant higher IgG prevalence of 38.8% than Garissa 35.9% and Tana River 32.2% (Chi square = 2.5, df = 2, *p* = 0.287). Males were significantly at higher risk of infection by RVFV than females (OR = 1.67, 95% CI 1.17–2.39, *p*<0.005). Age was significantly associated with RVFV infection (Wald Chi = 94.2, df = 5, *p*<0.0001). Individuals who had regular contact with cattle (OR = 1.38, 95%CI 1.01–1.89) and donkeys (OR = 1.38, 95%CI 1.14–1.67), or contact with animals through birthing (OR = 1.69, 95%CI 1.14–2.51) were significantly at a greater risk of RVFV infection than those who did not.

**Conclusion:**

This study demonstrated that although the Isiolo County has been classified as being at medium risk for RVF, virus infection appeared to be as prevalent in humans as in Tana River and Garissa, which have been classified as being at high risk. Populations in these counties live within nomadic livestock movement routes and therefore at risk of being exposed to the RVFV. Interventions to control RVFV infections therefore, should target communities living along livestock movement pathways.

## Author summary

Rift Valley fever (RVF) is a neglected mosquito-borne zoonotic disease that causes major outbreaks and economic harm to human and ruminants health leading to increased poverty within affected communities. RVF is caused by RVF virus (RVFV) affecting humans and a wide range of ruminants. The virus is transmitted through bites from mosquitoes and exposure to blood, body fluids, or tissues of infected ruminants. It was first isolated in Kenya in 1930 and several outbreaks have been recorded in many countries in sub-Saharan Africa. We studied pastoralist communities living along livestock migratory routes. Migratory livestock do move long distances in search of water and pasture and may be at higher risk of exposure to RVFV. We also determined risk factors for RVFV infection by studying age, gender, contact with animals through birthing, and occupation. Prevention and control of RVFV infection can target significant risk factors to prevent spread and re-occurrence of outbreaks.

## Introduction

Rift Valley fever (RVF) is an acute, mosquito-borne viral zoonotic disease of ruminants and humans causing outbreaks in Africa and Arabian Peninsula with significant negative public health and economic consequence [1]. The causing virus, RVF virus (RVFV) belongs to the family *Phenuiviridae*, genus *Phlebovirus*[2]. The virus was first isolated in Kenya in 1930 and extensively described in the 1931 epizootic among farmers and affected herds [3]. Since then, several RVFV outbreaks have occurred causing high morbidity and mortality in humans and livestock as well as significant economic loss in affected regions/countries [2]. The African continent has been affected mostly with human infections and outbreaks affecting Eastern Africa in 1997/98 and 2006/2007 which was widespread in Kenya, Tanzania, Somalia, Djibouti, Sudan and South Sudan [4]. The full impact of these outbreaks was not fully quantified but it was documented that Kenya suffered economic losses of up to US $ 32 million due to losses of animal herds, vaccination costs and trade bans [5]. In Kenya, the 1997/98 and 2006/2007 RVFV outbreaks led to 600 and 150 human deaths and over 27,000 and 700 estimated cases respectively, overstretching the limited public health resources and facilities in the North-Eastern regions of Kenya [5]. Other countries that have been affected include South Africa, Zimbabwe, Egypt, Mauritania, Senegal, Niger, Madagascar, Mayotte, Saudi Arabia and Yemen [6–10].

RVFV is transmitted by diverse species of mosquitoes broadly classified into primary vectors (floodwater *Aedes*) that maintain the virus for variable number of years in their drought resistant eggs deposited on wet soils on low lying depressions on land [11]. Following heavy persistent rains with flooding, the eggs hatch with a proportion already infected that emerge as infected adult female mosquitoes initiating transmission to nearby livestock which serve as amplifiers [12]. Subsequently, secondary vectors (*Culex* mosquitoes) are infected taking over transmission, potentially coupled with livestock and wildlife movement, and spread the virus far and wide resulting in outbreaks [13–15]. RVF mainly affects ruminants, especially cattle and sheep, and the transmission to humans is through direct contact with infected blood, animal tissue, abortus foetus, or birthing fluid, or through infected mosquito bites [16]. RVFV infection in livestock causes abortions and more than 95% perinatal mortality in livestock (i.e., sheep, goats, cattle, and camels) [10]. Most RVF cases in humans are subclinical with flu-like symptoms and may go undetected, but a small percentage of cases develop severe symptoms such as encephalitis and hemorrhagic fever disease with high case-fatality rates [17]. Ocular, liver, and kidney disease are also common complications [18, 19]. In addition, a significant association between RVFV infections during pregnancy and an increased risk for miscarriage in humans has recently been demonstrated [20]. Outbreaks are associated with large economic losses affecting agricultural production due to loss of livestock, vaccination costs, and trade ban on animals and animal products consequently resulting in poverty among communities which rely on livestock production as their economic mainstay [21].

Circulation of RVFV may expand worldwide due to demographic changes in the environment and climate variability in areas with close interaction between wildlife, livestock, vectors and human population [22–24]. Intense livestock production activities in close proximity to human populations and increased nomadic movement of livestock through areas with potential vector breeding sites during search of pasture and water, poses a threat of RVFV outbreaks to many countries [25]. Early warning systems are required in order to develop mitigation measures [26]. In previous outbreaks, late warnings, especially after rains and flooding, did not help to prevent massive economic losses [27].

Periodic outbreaks of RVF have occurred in Kenya, but limited information is available on the actual spatial distribution of infection among humans during the inter-epidemic period in areas associated with nomadic livestock movements. RVFV seroprevalence studies in humans have been performed in Kenya since 1987. The general RVFV seroprevalences have varied from 0% to 32%, depending on year of sampling and/or geographical location [4, 28]. In North-eastern Kenya there are many nomadic pastoral communities, and it has been shown that individuals with this lifestyle are at higher risk of infection with RVFV due to frequent contact with sick animals and animal products including blood, meat, and milk [29]. All counties in Kenya were recently classified into low, medium, or high risk for RVFV transmission based on the proportion of the national RVF epizootic years that the county has been involved in outbreaks since the first report of the virus in the district [12, 30, 31]. In the present study, sites occupied by pastoralist nomadic communities that fall in high and medium risk of RVFV transmission were selected.

The aims of this study were (i) to determine the extent of human exposure to RVFV infection in these zones with diverse ecologies, or communities, and (ii) to identify other contributing or confounding factors of infection. This is expected to provide an understanding of how the pastoral practices may cause human exposure to RVFV infection and influence disease circulation along animal movement and pastoral zones.

## Methods

### Study Sites

**Fig 1:**
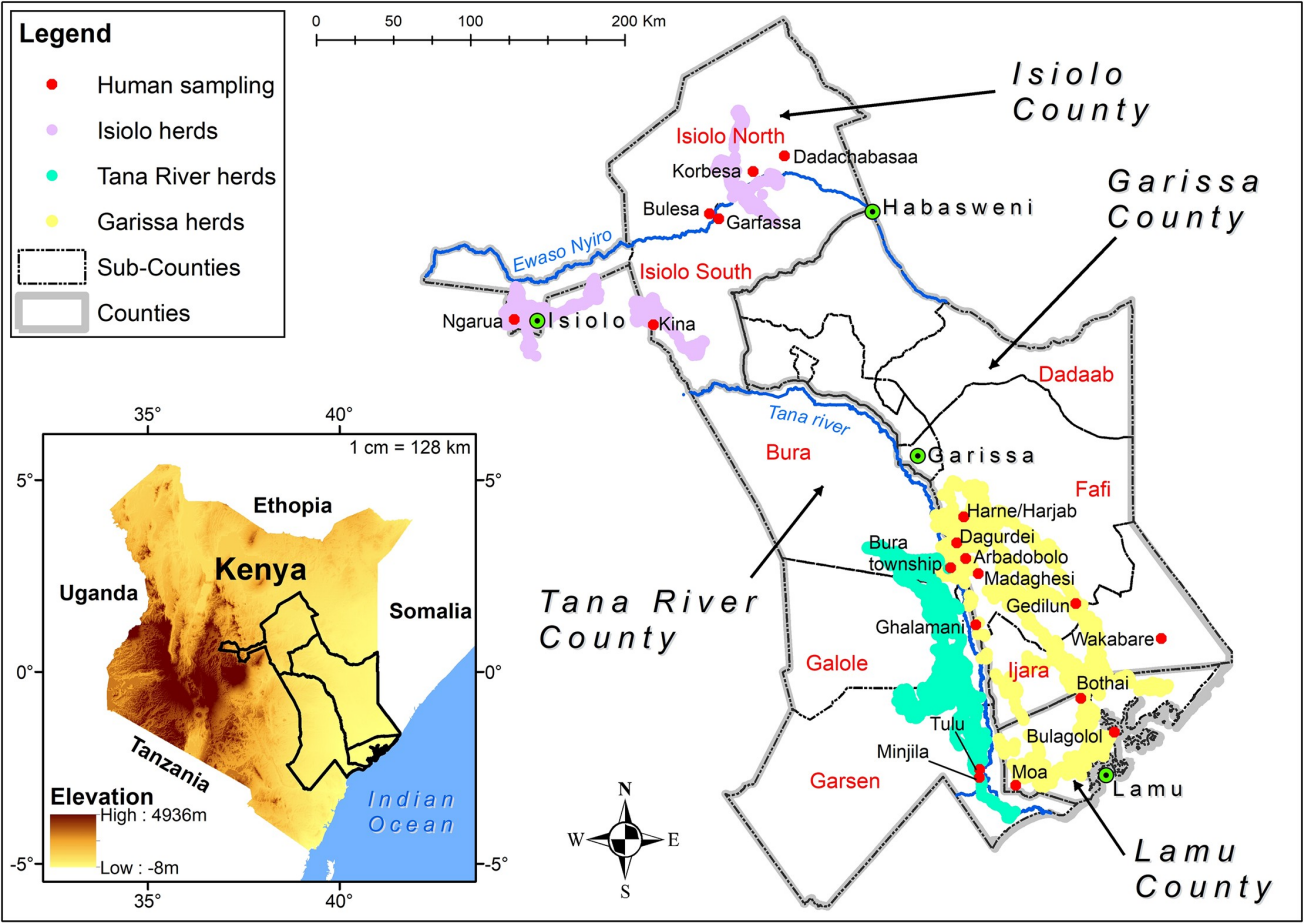
Map showing the study areas with specific villages samples indicated by red dots, towns indicated by green spots and herd movement routes in different colors as per the legend. (Source: 30m Digital Elevation Model from USGS was used to generate the insert map. County and Subcounty shapefiles were obtained from Kenya Open Data portal and the pastoralist migratory routes were monitored using GPS collar. The map was generated using ArcMap 10.2.2 from ESRI).

The study was carried out in arid and semi-arid RVF high-risk counties of Isiolo, Garissa, and Tana River, selected based on the RVF risk map and differential impact of the RVF outbreak in 2006/2007, where Garissa county had 300 human cases, Tana-River 16 human cases and Isiolo had 7 human cases (Fig 1) [31] ([32]. These areas are inhabited by nomadic pastoral communities who make use of this harsh environment by keeping sheep, goats, camels, and cattle as agricultural production is not feasible. The lifestyle of communities in these areas is characterized by periodic nomadic movement and temporary and/or permanent settlements in areas with sufficient pasture and water. The livestock migratory routes were monitored using GPS collar (Followit Sweden AB) installed on five selected herds in the three counties and their movement was monitored for two years. County and sub-county shapefiles were obtained from Kenya Open Data portal.

*Garissa County*: The Garissa study site (0° 27’ 25” S, 39° 39’ 30” E, 151 m elevation), has an annual mean temperature and rainfall of 28.8°C and 576 mm respectively, with high inter-annual inter-variability in rainfall. The vegetation in the area is mainly Acacia-Commiphora deciduous bush land and thicket (Savannah, Shrub land, open to very open shrubs) and on the coastal end lies the Boni Forest with indigenous open canopy forest that forms part of the Northern Zanzibar-Inhamdare Coastal Forest Mosaic. The site includes sandstones, dark clays and red sand soils. The site covers areas of Garissa, Ijara, and Lamu located between Tana River County and the boundary to Somalia (Fig 1). It has a population of 623,060 with most inhabitants from the Somali ethnic group. We sampled from Ijara, Fafi, Lamu East, and Lamu West sub-counties all in Garissa county. The main economic livelihood for the people living in Garissa is keeping nomadic livestock. About 90% of the population is directly dependent on livestock for daily nourishment and as a source of income. In Garissa, large livestock herds have to be moved during the dry season in search of pasture and water to the Tana River delta or into the Boni forest, which leads them through human settlements, wildlife and/or villages, different ecological zones (agro-pastoral, bushed low-lying grassland and dry humid ecological zones) and brings them in contact with wildlife (warthogs, gerenuk, waterbuck and buffalo) and different vector communities (*Aedes* and *Culex* mosquitoes). It is classified as a RVF high risk zone [30, 31] and is a major hotspot area in Kenya affected by the last outbreaks of RVF in 1997/98 and 2006/07 with over 300 cases reported [32, 33].

*Isiolo County*: Isiolo County (0° 21’ N, 37° 34’ 60 E, 1,145 m elevation) is within the arid to semiarid zone low-plains, characterized by infrequent rainfall, and predominantly flat with low-lying plains. Isiolo has an annual mean temperature of 26°C, an annual mean rainfall of 580 mm, and is served by the Ewaso Nyiro River, which is one of the main sources of water for both domestic and agricultural purposes. The Ewaso Nyiro flows through the county and partly bounds it. The county population is 143,294 (2009 census), divided into Isiolo North and Isiolo South sub-counties. It is inhabited predominantly by the Cushite communities (Oromo-speaking Boran and Sakuye), Turkana, Samburu, Meru and Somali. Apart from Meru, these communities’ practice nomadic pastoralism as a source of income. Livestock are moved for long distances during dry seasons in search of pasture and water encountering population settlements at different ecological zones, and vector communities predisposing them to risk of RVF infection. Isiolo County has been classified as a medium risk area for RVF. It was affected to a lesser extent by the outbreaks of 1997/98 and 2006/07 with 8 cases being reported [31].

*Tana River County*: The Tana River County borders to Garissa County to the Northeast (Fig 1). It lies between latitude 1° 7`S and 2° 3`S and longitude 40° 4`E and 41° 32`E, 1457 m elevation, and is divided into sub-counties including Garsen, Galole, and Bura. Rainfall is erratic, with rainy seasons in between March and May as well as October and December. The average annual rainfall is between 400mm and 750mm with a mean annual temperature ranging between 30°C and 33°C. It is sparsely populated with a population of about 240,075 according to the 2009 census [34]. The County is generally dry and prone to prolonged severe drought causing migration of nomadic livestock farmers to the Tana River delta where there are several areas of forest, woodland, and grassland where water and pasture is abundant. There are several wild ungulates that graze in the area (e.g., *Damaliscus lunatus topi*) as well as bird species. The major ethnic groups in this County are the Pokomos who practice agro-farming as well as the Orma and Wardey, who are predominantly nomadic livestock keepers. Tana River is an RVF hotspot affected by the 2006/7 RVF outbreak with 7 deaths and 16 reported probable cases [4]. The delta serves as a convergence zone for livestock, humans, and wildlife and a diversity of mosquito species including known vectors of RVFV that could facilitate transmission and exposure to RVFV infection. Flooding occurs, associated with occasional heavy rainfall in upstream areas of the Tana River.

### Study population

Randomized population sampling was used to select a pool of potential participants from 20 villages into the study and cross-sectional surveys were conducted between August 2014 and November 2015. A total of 1210 persons from the pool (male and female) aged ≥18 years living in villages at close proximity to the livestock movement routes were recruited into the study [35], (Fig 1), using the Cochran (1963) sample size calculation formula for prevalence studies [36]. The study subjects who consented to participate were randomly selected within their village of residence. A structured questionnaire was administered to collect socio-demographic data of the subjects after which blood samples were collected by venipuncture. Potential participant from the pool were excluded from the study if he or she did not consent to participate in the study.

### Ethical considerations

The study was approved by Kenya Medical Research Institute’s Scientific Ethics Review Unit (SERU) (SSC No. 2346). Informed consent was sought from all potential subjects and those meeting all inclusion criteria who consented to participate were recruited by signing a written informed consent. All personal information obtained in this study was kept confidential.

### Laboratory testing procedures

#### Analyses of IgM and IgG against RVFV

All serum samples were screened for exposure to RVFV using RVFV specific IgM and IgG enzyme-linked immunosorbent assays (ELISA). The commercial kits used were manufactured by Biological Diagnostic Supplies Limited (BDSL), Scotland, UK, and used according to the manufacturer protocols and published procedures. These kits were originally developed by the Special Pathogens Unit of the National Institute for Communicable Diseases (SPUNICD), Sandringham, South Africa [37]. The sensitivity and specificity to the standard panel of positive and negative samples was indicated as 100% respectively. Briefly, all serum samples were diluted 1:20 and heat inactivated at 56°C for 30 minutes and then the IgM and IgG ELISA were conducted per manufacturer instructions. The plates were analyzed at 405nm.

#### Interpretation of IgM and IgG ELISA results

Interpretations of results were done per the manufacturer instructions. The intensity of colors produced in the RVFV ELISAs were proportional to the amount of anti-RVFV IgG or IgM. The positive control optical density (OD) values ranged 0.81–1.7 for the test to be valid. Two intermediate OD values of the positive control were used for the calculation of the net mean OD value of the positive control (C++). The value was then used in the calculation of percentage positivity for the negative control (C-) and test serum using the formula as follows:

Percentage Positivity (PP) = Net OD serum (C-, or Test serum)

Net mean OD C++

Threshold PP value: Sera producing PP values ≥ 29 were considered to be positive and negative otherwise.

#### Plaque reduction neutralization test

Samples positive for RVFV antibodies by either IgM or IgG ELISA were further analyzed using a plaque reduction neutralization test (PRNT) to confirm the presence of antibodies against RVFV. Each virus isolate was diluted to a standard concentration that produced approximately 50 plaques. Serum samples were heat inactivated at 56°C for 30 min. Each sample was serially diluted in a sterile 96-well plate to determine the end point titer or highest dilution that neutralized at least 90% of the virus at 1:20 to 1:80 concentration in maintenance medium (minimum essential media [Sigma] with Earle's salts, 2% fetal bovine serum, 2% glutamine, 100 U/mL penicillin, 100 μg/mL streptomycin, and 1 μL/mL amphotericin B). A constant amount of diluted virus (1 x 10^8^ pfu/ml) was added into each well of the 96-well plate containing serially diluted serum samples and incubated for 1 h at 37°C. The virus–antibody mixture was then transferred to a 24-well plate with a confluent Vero cell monolayer and incubated at 37°C in CO_2_ for 1hr for virus adsorption, after which an overlay of 2.5% methylcellulose was added and incubated for 5–10 days at 37°C in CO_2_. The plates were retrieved from the incubator and stained with 0.25% crystal violet in absolute ethanol. All reactive sera from the initial screening at 1:20 were further serially diluted to determine the endpoint titer, in the highest dilution that neutralized 90% or greater of the RVFV relative to a serum-free control.

#### Explanatory variables

The explanatory variables included sex, age, and occupation of subjects, whether one had regular contact with cows, goats, donkeys, and whether contact with animals was made through birthing. We also controlled for site (Garissa = 1, Isiolo = 2, Tana river = 3).

### Analysis

All analyses were performed using Stata v13.1 (StataCorp, College Station, TX). First, we summarized the proportions positive for RVFV, and their 95% confidence intervals (CIs) estimated using Agresti-Coull method [38]. Next, we computed the intra-class correlation coefficient (ICC) to assess the variation in the outcome explained by clustering due to village of residence. The ICC measures the relatedness of the subjects within a group such as village and ranges from 0 (individuals within a group are as heterogeneous as individuals between groups) to 1 (members within a group show identical responses). It is the ratio of the variance component due to villages to the total variance for individual subjects[39], defined as

$$ICC=\sigma_{village}^{2}/\left(\sigma_{village}^{2}+\sigma_{subject}^{2}\right),$$

where $\sigma_{village}^{2}$ is the component of variance between villages and $\sigma_{subject}^{2}$ is the variance associated with subjects within villages. An ICC value of 0.027 was obtained, indicating that village of residence explained at least some variability in the outcome. Finally, to explore factors associated with RVFV infection while taking care of the correlations between outcomes from subjects in a village, we fitted a generalized estimating equations (GEE) model with a logit link function, assuming exchangeable working correlation [40]. Explanatory variables with a significant association (p<0.1) on univariable analyses were included in a backwards, stepwise regression model and rejected at the p≥0.05 significance level.

## Results

### Descriptive findings

Data were available on 1,210 individuals from 20 villages in three sites (Garissa, n = 664; Isiolo, n = 446; Tana River, n = 100). Sixty-two percent of the participants were females and the rest males. Their mean (median) age was 43.8 years (40.0 years). However, males were significantly older (49.8 years) than females (40.3, t-test *p*<0.0001). Most of them were pastoralists (47.7%), followed by housewives (26.6%), herdsmen (22.7%), crop farmer (1.8%), and the rest were classified as either teacher or student (1.2%). The participants reported to have regular contact with cattle (78.4%), goats (91.0%), and donkeys (30.3%). Approximately twelve percent reported to have made contact with animals through birthing.

### Prevalence of RVFV specific IgM and IgG, and risk factors for RVFV infection

Overall, 17 individuals (1.4%, 95%CI 0.8–2.3%) were IgM positive indicating an acute or recent RVFV infection. This proportion varied across the sub-counties, although not significantly (p = 0.830; Table 1). None was IgM positive in Isiolo South and Lamu west. We also observed that this proportion was similar in females (1.2%) and males (1.8%). The incidence was 1.6% among those who had contact with cattle and 0.8% among those who did not have contact with cattle. Among those who made contact with donkeys the incidence was 2.5%, and was below 1% among those who did not make contact with donkeys.

**Table 1:** Percent positive for Rift Valley fever virus by sub-county of residence.

Sub-county of residence		IgM		IgG	
	N	no. positive	% positive	no. positive	% positive
Isiolo County					
Isiolo North	240	5	2.1	118	49.2
Isiolo South	206	2	1.0	55	26.7
Garissa County					
Ijara	104	2	1.9	44	42.3
Fafi	316	6	1.9	104	32.9
Lamu East	167	1	0.6	63	37.7
Lamu West	77	0	0.0	26	33.8
Tana River County					
Garsen	100	1	1.0	31	31
Overall Prevalence	1210	17	1.4	441	36.4

N = total number of individuals sampled; no. = number

**Fig 2:**
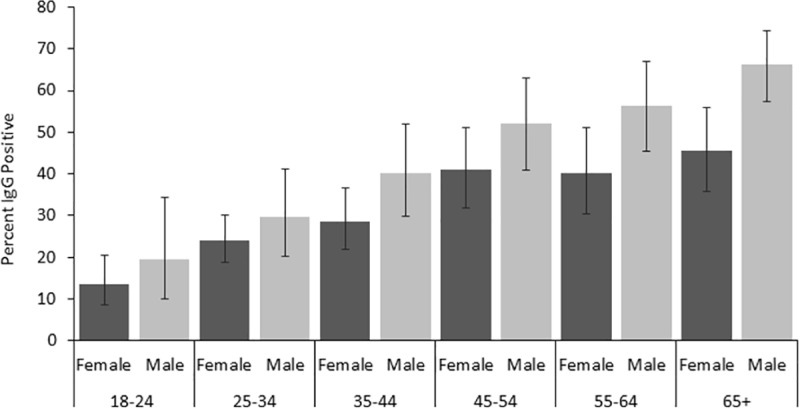
Rift Valley fever virus IgG prevalence by age group separately for females and males. Error bars indicate 95% confidence intervals.

Of 1,210 participants, 36.4% (95% CI 33.8–39.2%) were RVFV IgG seropositive by ELISA and neutralization assay. RVFV (IgG) prevalence was 35.7% in Garissa, 38.8% in Isiolo, and 31% in Tana River. These prevalences were not statistically different (Chi square = 2.5, df = 2, *p* = 0.287). There was a non-significant variation in RVFV exposure as measured by IgG, across occupation (Wald Chi = 0.98, df = 4, *p* = 0.913). Isiolo North, Ijara, and Fafi sub-counties (49%, 42% and 33%, respectively) had the highest prevalence of RVF compared to other sub-counties (Chi square = 8, df = 32, *p*<0.001; Table 1). The proportions RVFV IgG positive increased with age from 14.8% among the 18-24y old to 57.3% among those aged 65y and above (Fig 2).

Adjusted Odds ratios for RVFV IgG seropositivity using the GEE model are presented in Table 2. The results indicated that after adjusting for other factors, males were at a significantly 67% higher risk of infection by RVFV than females (aOR = 1.67, 95% CI 1.17–2.39). Increasing age was significantly associated with RVFV infection (Wald Chi = 94.2, df = 5, *p*<0.0001): compared to those aged 18–24 years, individuals in older age groups were two times more likely to have been exposed to RVFV infection (Table 2). After adjusting for other factors, those who made regular contact with cattle (*p* = 0.044) and donkeys (*p* = 0.001) were both at a significantly (38%) greater risk of RVFV exposure than those who did not (Table 2). Individuals who made contact with animals during birthing were also more likely to be infected with RVFV than those who did not (*p* = 0.009).

**Table 2:** Adjusted odds ratios for RVFV IgG seropositivity: generalized estimating equations model results.

Variable		Adjusted OR (95% CI)	*p*-value
Sex	Female	1	
	Male	1.67 (1.17–2.39)	0.005
County	Garissa	1	
	Isiolo	1.10 (0.68–1.77)	0.704
	Tana River	1.21 (0.71–2.07)	0.478
Age group	18–24	1	
	25–34	1.89 (1.30–2.75)	0.001
	35–44	2.57 (1.71–3.86)	< .001
	45–54	4.42 (2.78–7.01)	< .001
	55–64	4.72 (2.87–7.77)	< .001
	65+	6.72 (4.42–10.21)	< .001
Occupation	Pastoralist	1	
	Housewife	0.94 (0.61–1.45)	0.779
	Herdsman	1.04 (0.70–1.54)	0.857
	Teacher/student	0.78 (0.26–2.35)	0.653
	Crop farmer	1.33 (0.54–3.25)	0.532
Contact with cattle	No	1	
	Yes	1.38 (1.01–1.89)	0.044
Contact with donkeys	No	1	
	Yes	1.38 (1.14–1.67)	0.001
Contact by animal birthing	No	1	
	Yes	1.69 (1.14–2.51)	0.009

OR = odds ratio

## Discussion

This study analyzed human exposure to RVFV infection in villages along livestock migratory routes, which are convergence zones for the interaction between humans, animals, and the environment, reinforcing the One Health concept of RVF surveillance [41]. Acute infection in humans was noted in 17 (1.4%) asymptomatic individuals, measured by the presence of RVFV IgM. A large frequency, 36.4%, have had past exposure to RVFV measured by RVFV IgG. Significant risk factors included being male, older age, contact with cattle, contact with donkeys and contact with animals through birthing.

We monitored five herds that were found to move long distances in search of water and pasture. The Garissa county herd moved to Boni forest and back each year, passing through Fafi, Ijara, and Lamu sub-counties. The Tana River herds moved from Fafi and Bura sub-counties to the Tana Delta. The Tana River and Garissa county livestock were converging along the Tana River delta. The Isiolo herds moved long distances as well, with the Isiolo North and Isiolo South herds converging in some areas on their way back. The intense practice of pastoralism by these communities in search of pasture and water is a possible factor that could lead to increased exposure to RVFV infections as they move into new ecosystems, e.g. the Tana River delta. The climatic conditions in the three study sites are characterized by high temperatures and occasional flooding during the rainy season, favoring the emergence and survival of RVFV primary and secondary vectors that have the potential to cause outbreaks [10, 12, 15].

Data analyzed in the presented study were collected during 2014–2015, which was an inter-epidemic period, just before the 2015–2016 heavy El Niño rainfall alert that has previously been associated with RVF outbreaks. There was no large outbreak reported in 2016, and the high RVFV seroprevalence seen in the present study could potentially be a factor that reduced transmission and disease cases. Goats from two herds sampled during an RVF surveillance in Kenya in 2015/2016 in response to the El Niño rainfall alert showed RVF IgG antibodies confirming RVFV exposure [42]. Human infection with RVFV could in many cases be sub-clinical, since the seroprevalence reported generally in humans always exceeds the number of reported cases [28]. During outbreaks, majority of the cases that have clinical symptoms present with a mild febrile illness with no long-term sequelae and often go unreported [9]. We showed that the RVFV IgG prevalence for all three counties investigated was 36.4%. This is in the higher range compared to previous studies from Northeastern Kenya where the human RVFV seroprevalence was, for example, 15% in 1998 (Woods et al., 2002), 12.5–32% in 2006–2007 [4, 43–45], and 15–25% in 2009–2012 [1, 23, 46, 47]. Our findings showed males were at a significantly higher risk of infection by RVFV than females, and increased age was associated with higher RVF IgG prevalence. Furthermore, individuals who had regular contact with livestock had a greater risk of RVFV infection. Majority of the participants were females. Most males work away from home taking care of the livestock or on employment and hence were not available during sampling. Tana River had the least number of study participants due to inaccessibility during sampling arising from the insecure situation at the time of sampling. In terms of occupation, the majority were pastoralists and herdsmen which was expected as pastoralism is a major source of livelihood for populations living in Garissa, Isiolo, and Tana River counties.

The human RVFV seroprevalence was similar for all counties investigated. Isiolo County is regarded as a RVF medium risk County [31]. Our finding that RVFV infection, as measured by IgG, had similar prevalences in the neighboring counties could be attributed to the fact that the three counties share boundaries. Livestock movement across the counties usually occur hence the possibility of transmission of infection at convergence zones (watering and pasture points) like the Mboni Forest and Tana River delta, among others, as the animals are moved to pasture and water [35]. In addition, the presence of both primary and secondary vectors of RVFV (i.e., *Aedes mcintoshi* and some *Culex* species) could play an important role in the high prevalence observed in the study [12]. Similarly, in Garissa County, which borders Isiolo County, studies have shown an abundance of RVFV primary vectors (i.e. *Aedes mcintoshi* and *Ae*. *ochraceus*) as well as the secondary vectors (*i*.*e*., *Culex spp*) known to transmit RVFV during floods [11, 12, 48]. Another factor is the human behavior such as handling of sick animals, sheltering of animals, slaughtering practices, and birthing, that could differ across the counties and could influence the risk of infection [49, 50].

This study showed that males were at a significantly higher risk of past exposure to RVF due to increased IgG prevalence than females. We speculate that men spend more time outdoors in contact with animals as they are herding, and exposure may have happened over time. This increases the risk of exposure to infectious mosquito bites since they usually do not have adequate protection. This is consistent with the findings by Anyangu et al., 2010, which reported a high proportion of acute RVFV infection and severe disease in males [16]. Similarly, the occupation of herding and pastoralism is dominated by males, which involves high risk animal-related activities like consuming or handling sick animal products, sheltering sick animals at home and away, birthing, slaughtering, milking, or skinning of dead or sick animals [23]. On the contrary, due to lack of knowledge, women get infected as they take care of sick animals at home hence posing a significant risk of infection to them especially for rural women [50]. RVFV prevalence was lower among the younger age groups [1, 29]. Another study conducted in Kenya also observed that there was a significant association of RVFV seropositivity with age and that the odds of RVFV seropositivity in the elderly was twice that of the younger age group[45]. In our study, increasing age was significantly associated with RVFV infection. This can be attributed to the cumulative effect of exposure over time and the possibility of the older population having been exposed to RVFV infected mosquitoes, sheltering of sick animals, slaughtering infected animals, contact with infected arbutus, disposal of infected fetus or previous exposure during the major outbreak in 1997/98 and 2006/2007 in these regions [4, 10, 23]. We speculate that the younger age-group are also less likely to handle these infectious materials and hence the reduced likelihood of exposure. It is therefore possible that the high RVFV prevalence observed in all the sites between the year 2014 and 2015 could be due to the past large outbreaks experienced in Kenya in 1996/97 and 2006/2007 [1, 29].

RVF can infect many species of animals causing severe disease in domesticated animals including cattle, sheep, camels and goats. Sheep and goats appear to be more susceptible than cattle or camels [22]. Donkeys were included in this study because they are domesticated in large numbers in the study area and mosquito blood meal analysis have shown donkey blood making donkeys a possible reservoir for RVFV that should be looked into. We found out that persons who made regular contact with cattle and donkeys were at a significantly greater risk of RVFV infection than those who did not, a finding which was also observed by a study conducted by Anyangu et al, 2010, during the 2007 outbreak in Kenya [16]. This can be attributed to the fact that the animals they come in contact with through high risk related activities mentioned earlier could be infected with RVFV hence transmission. Animal contact can occur through slaughtering, herding, sheltering sick animals within the homestead, birthing, or consuming and handling products from sick animals such as milk, meat, and blood [10, 51]. Our study has shown that participants with regular contact with animals through birthing were at higher risk of RVFV infection. Community education should be done on these risk factors to minimise disease transmission.

Although Isiolo is classified as being at medium risk of RVF outbreaks, and Garissa and Tana River as being at high risk [31], our study revealed no significant difference in seroprevalence among the three counties. This is contrary to the expectation based on the risk level on the proportion of the national RVF epizootic years that the counties were involved in, since the first report of the virus in the district [31]. The risk of transmission through exposure to infected animals and animal products could be low in Isiolo County, because the IgG seroprevalence among livestock in the same area during the 2006/2007 outbreak was much lower in Isiolo (9.5%) compared to Garissa (20.9%) county[52]. We however cannot rule out that differences in human behavior such as use of personal protective equipment during birthing, slaughtering, handling of sick animals, among others, that could expose the population in Isiolo county to a higher risk of infection [49, 50]. Interestingly, reports from the last outbreaks did not document any human deaths in Isiolo County [4]. Human cases or deaths are known to occur after exposure to infected animals or animal products or fluids. In our case, we can speculate that although there were no severe human cases reported during the outbreak in 2006 [4], human exposure might have occurred through bites from infectious mosquito that resulted in mild to subclinical infection responsible for the observed increased seroconversion. The vector species composition observed in Isiolo by previous studies showed high densities of secondary vectors which could be associated with human transmission and the complete absence of a key primary vector *Aedes ochraceous* [12] may have a bearing on the livestock transmission.

Considering these findings, we strongly recommend that animals moving along these routes be vaccinated during RVF outbreak alerts in response to early warning signs to protect the populations at risk. Ongoing efforts to develop human RVF vaccine should target these populations as important beneficiaries. Continual stake holder engagement will be done during dissemination of findings to sensitise communities and community leaders on infection control measures when handling animals and meat products. This includes decreased contact with blood, body fluids, or tissues of infected animals by wearing personal protective equipment. In addition to these, communities can protect themselves against mosquito bites by use of mosquito repellents and bed nets. Public health officials will use these findings to provide information regarding suggested protective and infection control measures as well as sustainable environmental monitoring and case surveillance systems that will aid in the prediction and control of RVF outbreaks.

An important limitation of this study is that we did not sample a comparison or control group comprising people that do not live in the nomadic pastoral zones because of funding limitation to be able to travel and access all areas. However, the study still managed to generate valuable information of RVFV exposure in age cohorts of humans in RVF-prone areas.

## Conclusion

This study established that a large proportion of populations living in the three Counties of Garissa, Isiolo, and Tana River have been exposed to RVFV infection. The significant risk factors associated with RVFV infection include age, being male, contact with cattle and donkeys, and contact with animals through birthing. Infection control measures should target these significant risk factors to reduce disease transmission in the area. The data generated by this study will be useful to public health officials in determining the disease risk levels of individuals living along the animal movement routes and in designing appropriate targeted infection control measures to prevent epidemics.

### Other information

We thank the staff of ICIPE, MLEID laboratory for hosting the laboratory work. We would like to thank all the study participants for donating samples, the Ministry of Public Health and Sanitation staff, and the community leaders in the three project sites for the support they offered in sample collection.

## Supporting information

S1 Checklist (DOC)
