# Supplementary material for: High risk for human exposure to Rift Valley fever virus in communities living along livestock movement routes: A cross-sectional survey in Kenya
Source: PLoS Negl Trop Dis. 2020 Feb 21;14(2):e0007979. doi: 10.1371/journal.pntd.0007979 (PMC7055907; doi:10.1371/journal.pntd.0007979)
Supplement: S1 Checklist — (DOC) [file pntd.0007979.s001.doc]

STROBE Statement—Checklist of items that should be included in reports of ***cross-sectional studies***

|  | Item No | Recommendation |
| --- | --- | --- |
| **Title and abstract** | 1 | (*a*) Indicate the study’s design: **Cross-sectional survey - mentioned in the title** |
|  |  | (*b*) Provide in the abstract an informative and balanced summary of what was done and what was found – **Introduction, methods, results and conclusion provided** |
| Introduction | | |
| Background/rationale | 2 | Explain the scientific background and rationale for the investigation being reported – **Introduction: Paragraphs 3 and 4** |
| Objectives | 3 | State specific objectives, including any prespecified hypotheses: **Introduction, Paragraph 5** |
| Methods | | |
| Study design | 4 | Present key elements of study design early in the paper – **Methods section; Page 11, Paragraph 1.** |
| Setting | 5 | Describe the setting, locations, and relevant dates, including periods of recruitment, exposure, follow-up, and data collection: **Methods, Page 6 to 10 under study sites** |
| Participants | 6 | (*a*) Give the eligibility criteria, and the sources and methods of selection of participants - **Methods; Page 11, Paragraph 1.** |
| Variables | 7 | Clearly define all outcomes, exposures, predictors, potential confounders, and effect modifiers. Give diagnostic criteria, if applicable – **Data analysis page 13 and 14- Paragraph 1** |
| Data sources/ measurement | 8* | For each variable of interest, give sources of data and details of methods of assessment (measurement). Describe comparability of assessment methods if there is more than one group - **Pages 11-13;** **Data analysis page 14- Paragraph 1** |
| Bias | 9 | Describe any efforts to address potential sources of bias – **Study population subsection, paragraph 1: Randomized population sampling was used;** **Data analysis section, page 13 and 14- Paragraph 1, adjustment for cluster effect and explanatory variables in a multivariable analysis.** |
| Study size | 10 | Explain how the study size was arrived at – **Under Study population – page 11 – paragraph 1** |
| Quantitative variables | 11 | Explain how quantitative variables were handled in the analyses. If applicable, describe which groupings were chosen and why. **Data analysis section, Pages 13-14.** |
| Statistical methods | 12 | (*a*) Describe all statistical methods, including those used to control for confounding – **Data analysis section, paragraph 1 – pages 13-14.** |
|  |  | (*b*) Describe any methods used to examine subgroups and interactions - **Data analysis section, paragraph 1 – page 13 and 14.** |
|  |  | (*c*) Explain how missing data were addressed – **N/A** |
|  |  | (*d*) If applicable, describe analytical methods taking account of sampling strategy - **N/A** |
|  |  | (*e*) Describe any sensitivity analyses - **N/A** |
| Results | | |
| Participants | 13* | (a) Report numbers of individuals at each stage of study—eg numbers potentially eligible, examined for eligibility, confirmed eligible, included in the study, completing follow-up, and analysed – **Results – page 14 to 16** |
|  |  | (b) Give reasons for non-participation at each stage - **N/A** |
|  |  | (c) Consider use of a flow diagram – **Data summarized in a table – see table 1** |
| Descriptive data | 14* | (a) Give characteristics of study participants (eg demographic, clinical, social) and information on exposures and potential confounders – **Descriptive findings section, paragraph 1** |
|  |  | (b) Indicate number of participants with missing data for each variable of interest - **None** |
| Outcome data | 15* | Report numbers of outcome events or summary measures – **Page 15, paragraphs 1 and 2** |
| Main results | 16 | (*a*) Give unadjusted estimates and, if applicable, confounder-adjusted estimates and their precision (eg, 95% confidence interval). Make clear which confounders were adjusted for and why they were included – **Results section, pages 15 to 16.** |
|  |  | (*b*) Report category boundaries when continuous variables were categorized. **See Table 2 for age.** |
|  |  | (*c*) If relevant, consider translating estimates of relative risk into absolute risk for a meaningful time period. **N/A** |
| Other analyses | 17 | Report other analyses done—eg analyses of subgroups and interactions, and sensitivity analyses.**N/A** |
| Discussion | | |
| Key results | 18 | Summarise key results with reference to study objectives: **Discussion on page 18** **Paragraph 1** |
| Limitations | 19 | Discuss limitations of the study, taking into account sources of potential bias or imprecision. Discuss both direction and magnitude of any potential bias |
| Interpretation | 20 | Give a cautious overall interpretation of results considering objectives, limitations, multiplicity of analyses, results from similar studies, and other relevant evidence - **Conclusion – page 22, paragraph 1** |
| Generalisability | 21 | Discuss the generalisability (external validity) of the study results: **Conclusion – page 22, paragraph 1** |
| Other information | | |
| Funding | 22 | Give the source of funding and the role of the funders for the present study and, if applicable, for the original study on which the present article is based – **Page 23, Paragraph 2** |

*Give information separately for exposed and unexposed groups.

**Note:** An Explanation and Elaboration article discusses each checklist item and gives methodological background and published examples of transparent reporting. The STROBE checklist is best used in conjunction with this article (freely available on the Web sites of PLoS Medicine at http://www.plosmedicine.org/, Annals of Internal Medicine at http://www.annals.org/, and Epidemiology at http://www.epidem.com/). Information on the STROBE Initiative is available at www.strobe-statement.org.
